# Antibacterial Agent-Loaded, Novel In Situ Forming Implants Made with Poly(Isosorbide Sebacate) and Dimethyl Isosorbide as a Solvent for Periodontitis Treatment

**DOI:** 10.3390/molecules30244717

**Published:** 2025-12-09

**Authors:** Monika Śmiga-Matuszowicz, Bożena Nowak, Danuta Wojcieszyńska

**Affiliations:** 1 Department of Physical Chemistry and Technology of Polymers, Silesian University of Technology, M. Strzody 9, 44-100 Gliwice, Poland; 2 Institute of Biology, Biotechnology and Environmental Protection, Faculty of Natural Sciences, University of Silesia in Katowice, 40-032 Katowice, Poland

**Keywords:** isosorbide, poly(isosorbide sebacate), in situ forming implants, antibacterial activity, periodontitis

## Abstract

Isosorbide-based aliphatic polyesters are a promising class of biodegradable polymers for biomedical applications, representing an attractive alternative to poly(α-hydroxy acids). Derived from the bio-based bicyclic diol, they combine structural rigidity, tunable hydrophilicity, and enhanced biocompatibility, making them suitable for drug delivery and sustainable medical devices. In this study, we developed novel in situ forming implant (ISFI) formulations composed of poly(isosorbide sebacate) (PISEB) and dimethyl isosorbide (DMI), and evaluated their applicability for local delivery of doxycycline hyclate (DOXY), minocycline hydrochloride (MIN), and/or eugenol (EUG). Basic characteristics of new ISFI formulations were investigated. Rheological analysis demonstrated that the liquid formulations exhibited shear-thinning behavior, which is advantageous for ISFI systems. However, the MIN-loaded formulation exhibited excessively rapid drug release, with a pronounced initial burst (86.4 ± 5.9%) within 24 h, whereas the DOXY-loaded system showed a lower burst of 41.1 ± 5.9% over the same period. The effect of EUG addition on depot morphology and antibiotic release profiles was also assessed. In vitro drug release studies demonstrated that EUG reduced the release rate of both antibiotics, increasing and prolonging their antibacterial activity. Eugenol co-released with antibiotics also reduced the pro-inflammatory effect of the released antibiotic doses by more than tenfold.

## 1. Introduction

Periodontal diseases are among the most common microbial infections, affecting up to 90% of the adult population worldwide [1,2]. They are generally classified into two main types: gingivitis and periodontitis. Gingivitis, the milder form, is characterized by gum swelling and bleeding during brushing. Although it is easily reversible with proper oral hygiene, it can progress to a chronic condition known as periodontitis [1,3]. Periodontitis, in turn, is a chronic, inflammatory disease of the tooth-supporting tissues that leads to progressive destruction of the surrounding soft and bone tissues. This progression results in the formation of periodontal pockets that provide a favorable environment for pathogenic microorganisms, which, in turn, leads to subsequent tooth mobility and loss.

Effective treatment for periodontitis depends not only on the selection of an appropriate antimicrobial agent but also on its route of administration. Systemic antimicrobial therapy can be effective, but it requires high doses to achieve therapeutic concentrations in the periodontal pocket [4]. In clinical practice, local drug delivery systems are often preferred over systemic therapy because they provide higher drug concentrations at the site of infection while minimizing adverse effects [5,6]. Local drug delivery systems administered directly into the periodontal pocket are capable of maintaining effective concentrations of active agents for the required duration. Among these, in situ forming implants (ISFIs) represent a convenient option, as these liquid formulations can be readily injected using a standard syringe and needle. Once administered, they solidify into a depot at the site, ensuring sustained release of therapeutic agents within the periodontal pocket. Consequently, ISFIs provide high drug concentrations at the site of infection while simultaneously minimizing systemic exposure and adverse effects.

ISFI systems generally consist of three primary components: (1) a water-insoluble, biodegradable polymer, (2) a non-toxic, water-miscible organic solvent capable of dissolving the polymer, and (3) a therapeutic agent, which may be incorporated either in dissolved or suspended form within the polymer solution. A representative example of this technology, developed by Dunn and marketed under the trade name ATRIGEL^®^, involves the addition of the drug to the polymer solution just before injection [7]. By modulating the formulation composition: selection of polymers, solvents, and specific additives, ISFIs can be adapted to provide controlled drug release ranging from several days to several months [8,9,10].

Currently, the majority of commercially available ISFI systems are based on aliphatic polyesters, such as polylactide (PLA) and poly(lactide-co-glycolide) (PLGA), combined with N-methyl-2-pyrrolidone (NMP) as the solvent. However, it has been reported that the heterogeneous hydrolytic degradation of aliphatic polyesters may cause a pronounced decrease in pH within the polymeric core, mainly due to the high solubility and low pKa values of lactic acid and glycolic acid (pKa 3.86 and 3.83, respectively). In fact, some studies have documented local pH values as low as 1.5 during the degradation of these polymers [11]. The accumulation of acidic degradation products in high concentrations can adversely affect implant biocompatibility by eliciting inflammatory tissue responses and compromising the stability of therapeutic agents, particularly peptides and proteins [12]. This issue becomes especially critical when degradation occurs in anatomical sites with limited fluid exchange [13]. That is why biodegradable polyesters derived from renewable raw materials, such as polysuccinates or polysebacates, appear to be a promising alternative to substitute for PLA and PLGA in selected biomedical applications.

Therefore, in the present study, we propose isosorbide-based polysebacate (PISEB) and the isosorbide-derived solvent dimethyl isosorbide (DMI) as the core components of novel ISFI formulations. To the best of our knowledge, this is the first report describing the use of isosorbide both as the polymer and solvent component in the development of ISFI-based drug delivery systems.

Isosorbide (IS; 1,4:3,6-dianhydro-D-sorbitol) is one of three stereoisomers of 1,4:3,6-dianhydro-D-hexitols derived from biomass. Among them, IS is the most readily available, as it is obtained directly from sorbitol. IS has a molecular structure composed of two cis-fused tetrahydrofuran rings forming a 120° angle and two secondary hydroxyl groups. Owing to the presence of hydroxyl groups, IS can serve as a diol monomer in polyester synthesis or be further modified into various low-molecular derivatives [14,15,16]. Due to its bio-based origin, non-toxic profile, rigid bicyclic structure, and physicochemical properties, IS is an attractive candidate for polyester production. In our previous studies, isosorbide-based compounds have been explored for a range of biomedical applications. Unsaturated IS derivatives have been proposed as polymerizable components of injectable, stable, or biodegradable bone fillers in a physiological environment [17,18]. Additionally, aliphatic IS-based polyesters, including polysuccinates and polysebacates, have been investigated as components of injectable local drug delivery systems [19,20] as well as biodegradable electrospun scaffolds for vascular regeneration [21]. Importantly, IS has been confirmed to be non-toxic in absorption, distribution, metabolism, and excretion (ADME) studies in rats [22]. After administration, IS demonstrated rapid absorption and elimination, with a bioavailability of 96.1%. Metabolic stability assays further confirmed that IS is not metabolized, with the majority of the absorbed compound excreted via urine.

Dimethyl isosorbide (DMI) is a non-toxic, water-soluble, polar solvent with a high boiling point (235–237 °C at 1013 hPa), derived from lignocellulosic biomass through sorbitol and isosorbide intermediates [23,24]. Owing to its properties, DMI has been successfully applied in various fields, including sustainable membrane fabrication [25,26], biomass pretreatment and downstream processing [27], and polyethylene terephthalate dissolution [28]. In addition to its industrial uses, DMI is also used in biomedical and cosmetic applications. It has been incorporated into skin care formulations and transdermal therapeutic systems, where it serves as a safe penetration enhancer [29,30]. Moreover, DMI has proven valuable as a solvent carrier for pharmaceuticals due to its ability to maintain drug stability. For instance, it has been used in liquid aspirin formulations, where other solvents fail to ensure sufficient stability [31,32]. The low toxicity of DMI was confirmed in multiple toxicological studies [33,34].

Effective therapy against periodontitis relies not only on the design and composition of the implant, but also on the careful selection of therapeutic agents. The choice of active compounds is critical to ensure adequate antimicrobial activity at the site of infection.

Tetracyclines are bacteriostatic antibiotics widely employed in clinical practice due to their broad-spectrum activity against aerobic and anaerobic, Gram-positive and Gram-negative bacteria. Their primary mechanism of action involves binding to the bacterial 30S ribosomal subunit, thereby preventing tRNA from attaching to the acceptor site of the ribosome-mRNA complex. This interaction disrupts bacterial protein synthesis, resulting in the inhibition of growth and replication.

Minocycline (MIN, 7-dimethylamino-6-dimethyl-6-deoxytetracycline) is a second-generation, semi-synthetic tetracycline analog that has been in use for more than four decades. Beyond its antimicrobial properties, MIN at concentrations detected in gingival crevicular fluid has been shown to stimulate osteoblast activity, while prolonged exposure of these cells to tetracyclines results in a proportional increase in mineralized bone matrix [35,36]. Another member of this class, doxycycline hyclate (DOXY, 6-deoxy-5-hydroxytetracycline hydrochloride hemihydrate hemiethanolate), also exhibits additional therapeutic benefits. It inhibits the activity of matrix metalloproteinases (MMPs), enzymes implicated in collagen degradation and the progression of periodontal disease [37,38]. Given their proven efficacy, minocycline and doxycycline have therefore been incorporated into commercially available systems designed for local periodontal therapy [5,6].

In recent years, the number of antibiotic-resistant microbial strains has grown substantially [39]. One of the key factors enabling bacteria to survive even in the presence of high antibiotic concentrations is their ability to form biofilms [40]. As a result, increasing attention has been given to the development of formulations containing not a single but multiple active substances, which synergistically reduce the pathogen’s capacity to persist within the host environment. Among such adjuvant compounds used in dentistry, special attention has been given to the plant-derived eugenol (EUG), a principal constituent of plant essential oils of the *Lamiaceae*, *Lauraceae*, *Myrtaceae*, and *Myristicaceae* families. For example, EUG is the major component of clove (*Syzygium aromaticum*) essential oil, accounting for 85–95% of its content [41]. Chemically, EUG (4-allyl-2-methoxyphenol) is a phenylpropanoid formally derived from guaiacol, with an allyl chain at the para position relative to the hydroxyl group. Numerous studies have demonstrated that EUG directly targets microbial cellular processes, including gene expression levels, and disrupts microbial quorum-sensing (QS) communication, thereby reducing the risk of resistance development [42,43]. In addition to its antimicrobial activity, EUG exhibits anti-inflammatory, analgesic, and antioxidant properties and is used topically on the gums to reduce microbial load and alleviate discomfort during dental procedures. The World Health Organization (WHO) has classified EUG as a generally recognized safe substance. However, when incorporated into ISFI systems, the volatile nature and limited aqueous solubility of eugenol may influence the release kinetics of active agents from the implant into surrounding tissues, thereby modulating the antimicrobial activity at the diseased site.

The present study aimed to (I) characterize and evaluate novel ISFI formulations prepared from poly(isosorbide sebacate) (PISEB) and the isosorbide-based solvent dimethyl isosorbide (DMI); (II) assess the release profiles of doxycycline (DOXY), minocycline (MIN), and eugenol (EUG) into phosphate-buffered saline (PBS) from the precipitated implants; (III) determine the antibacterial activity of the formulations; and (IV) evaluate their anti-inflammatory potential.

## 2. Results

### 2.1. Preparation and Rheological Behavior of PISEB-Based Liquid Formulations

The liquid formulations were prepared from a mixture of PISEB and DMI. The polymer was dissolved in DMI at ambient temperature for 24 h, yielding a clear solution with a concentration of 20%. In selected formulations, 10, 30, or 50 wt.% of DMI was replaced by EUG. One of the antibiotics, DOXY or MIN, was added to the formulations just before injection into PBS. Since neither antibiotic is soluble in DMI or EUG, these formulations were suspensions of the drug in polymer solution. The chemical structures of all components used in the preparation of ISFI formulations are shown in Figure S1 (Supplementary Information). The detailed compositions of prepared ISFI formulations are summarized in Table 1.

**Figure 1:**
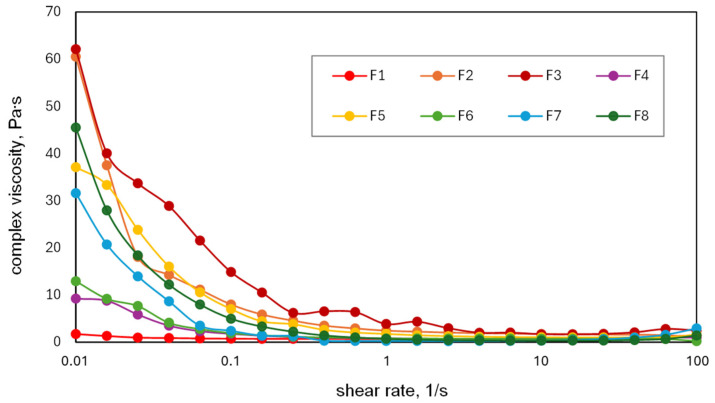
Complex viscosity versus shear rate for PISEB-based formulations tested at 25 °C (n = 3).

ISFI systems are designed to be administered by injection using a syringe and, therefore, their viscosity must fall within an acceptable range to enable efficient injection through standard needles. The viscosity should be sufficiently low to allow easy injection, but also high enough to stabilize the suspension of insoluble drugs and ensure controlled drug release [8]. To evaluate these properties, flow experiments were conducted for all liquid formulations. The complex viscosity versus shear rate was measured using a parallel plate rheometer at 25 °C. The resulting viscosity–shear rate curves are shown in Figure 1.

As shown in Figure 1, the viscosity of almost all formulations, regardless of composition, decreases with increasing shear rate, indicating a non-Newtonian shear-thinning behavior. This effect was the least pronounced for formulations F4 and F6, which have relatively low viscosities at 0.01 s^−1^ (12.9 and 9.2 Pa·s, respectively). In contrast, formulation F1 (PISEB solution in DMI) exhibited Newtonian flow behavior with viscosity maintained in the range of 1.0 to 1.5 Pa·s, independent of shear rate. The decrease in viscosity with the increase in shear rate was significant for the formulations containing EUG (F3, F5, F7, and F8) and DOXY (F2, F3) or MIN (F5). Increasing the proportion of EUG from 10 to 50 wt.% of solvent led to a marked rise in viscosity. A similar effect was observed with the addition of DOXY or MIN, which were present as solid particles suspended in polymer solutions. The incorporation of DOXY (F2 and F3) resulted in a four-fold increase in viscosity compared with F6, and nearly a forty-fold increase compared with F1. However, at shear rates above 1 s^−1^, their viscosities declined to comparably low levels.

### 2.2. Morphology of the Implants

**Figure 2:**
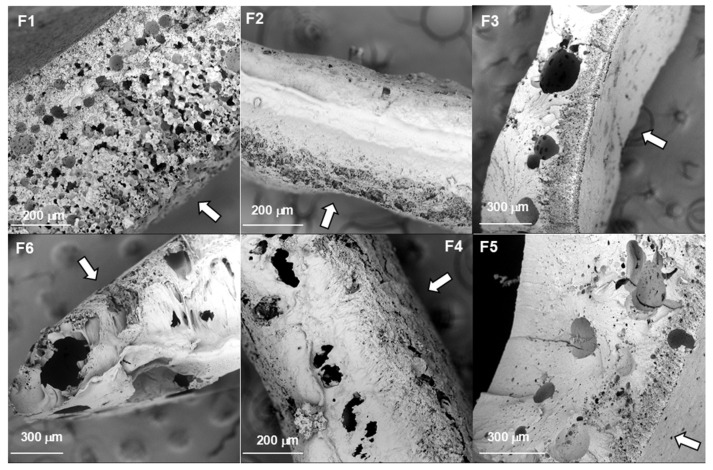
Representative scanning electron microscopy (SEM) images of the lyophilized depot cross-sections after 24 h. F1 (unloaded implant), F2 (DOXY), F3 (DOXY + EUG), F4 (MIN), F5 (MIN + EUG), F6 (EUG, 10%). The arrows indicate the upper surface of the implant.

**Figure 3:**
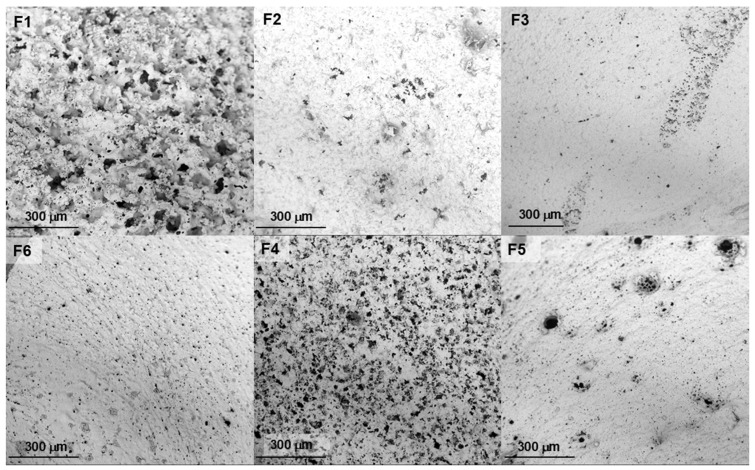
Representative scanning electron microscopy (SEM) images of the lyophilized depot’s surface after 24 h. F1 (unloaded implant), F2 (DOXY), F3 (DOXY + EUG), F4 (MIN), F5 (MIN + EUG), F6 (EUG, 10%).

To evaluate the microstructure of the precipitated depots, SEM observation was performed on both surface and cross-sectional samples. Figure 2 and Figure 3 show the SEM images of the depot’s morphology after 24 h from injection into PBS. It can be seen that the cross-sectional surface of most depots appears comparable, except depot F1, which is composed exclusively of PISEB and DMI. The implant F1 exhibited a more porous architecture, both within the internal structure and across the surface (Figure 2F1 and Figure 3F1).

The incorporation of EUG in the formulations leads to the formation of depots with a more uniform and denser surface layer. However, underneath this thin outer skin, a more porous sublayer with a thickness of about 100 μm was observed. Based on the SEM cross-sectional images of implants (Figure 2), it was possible to assess their macroscopic shape. Upon injection into PBS, all formulations formed spherical depots, which flattened within minutes. The thickness of the plane implants was estimated to range between 500 and 800 μm. The morphology of the upper surface of implants is presented in Figure 3. The surface layer of the depots containing EUG (F3, F5, F6) and DOXY (F2) was smooth and dense, with few pores. In turn, the surface of the depot containing MIN (F4) was less porous compared to F1 but more porous than the other formulations.

### 2.3. In Vitro Drug Release

**Figure 4:**
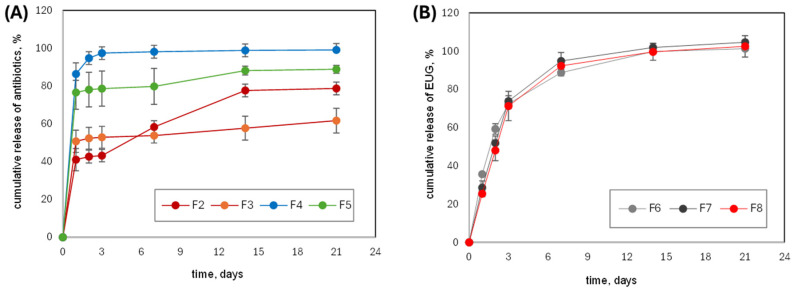
Cumulative amount of (**A**) antibiotics and (**B**) eugenol released (as a percentage of the amount initially loaded into the implant) from PISEB-based depots as a function of immersion time in PBS (n = 3).

In this investigation, PISEB-based formulations were loaded with two selected antibiotics, DOXY or MIN, and their release profiles from the formed depots in PBS medium (pH 7.4; 37 °C) were monitored over a period of 21 days. The concentration of the released antibiotics in PBS was determined using UV-VIS spectrophotometry. As shown in Figure 4A, the composition of the ISFI formulation was a key parameter determining the release behavior of both antibiotics from the formed depots.

In the case of DOXY, the presence of EUG (formulation F3) enhanced the initial burst release of the antibiotic; however, after 3 days, the release rate decreased compared to DOXY alone (formulation F2). In turn, EUG significantly reduced the release of MIN (formulation F5) throughout the entire 21-day period and also decreased the initial burst release during the first 24 h. A comparison of both antibiotics indicates that DOXY was released more slowly than MIN, with approximately 63% and 80% of the loaded drug released from the F3 and F2 depots, respectively, over 21 days.

Since EUG is also a therapeutic substance, it was reasonable to investigate its release profile from PISEB-based formulations without antibiotics. Figure 4B presents the EUG release curves for three different formulations containing 10% (F6), 30% (F7), or 50% (F8) EUG partially replacing the DMI solvent. As illustrated in Figure 4B, the cumulative release profiles of EUG are highly comparable, regardless of the proportion of EUG in the formulation.

To assess the kinetics of drug release, several mathematical models were applied to the experimental data. The most appropriate kinetic model was selected based on linearity and the correlation coefficient (r^2^), which statistically indicates how well the data fit the individual model. The r^2^ values and rate constants for each model are summarized in Table 2.

The results showed that, for the formulations containing EUG (F6, F7, F8) without antibiotics, the first-order model provided the best fit (highest r^2^) compared with the other models. For two DOXY-containing formulations (F2 and F3) and formulation F5 (MIN with EUG), the Higuchi model exhibited the strongest linear correlation with the experimental data. In contrast, the release data for formulation F4, which contained MIN, did not fit any kinetic model well, as the r^2^ value in each case was below 0.6. A probable explanation could be the rapid release of MIN from the F4 formulation, with an enormous burst release in the first 24 h (86% of the loaded MIN). Representative examples of release data for F2 and F6 implants, fitted to various kinetic models, are presented in Figure S2A,B (Supplementary Information).

### 2.4. In Vitro Antibacterial and Inflammatory Potential

**Figure 5:**
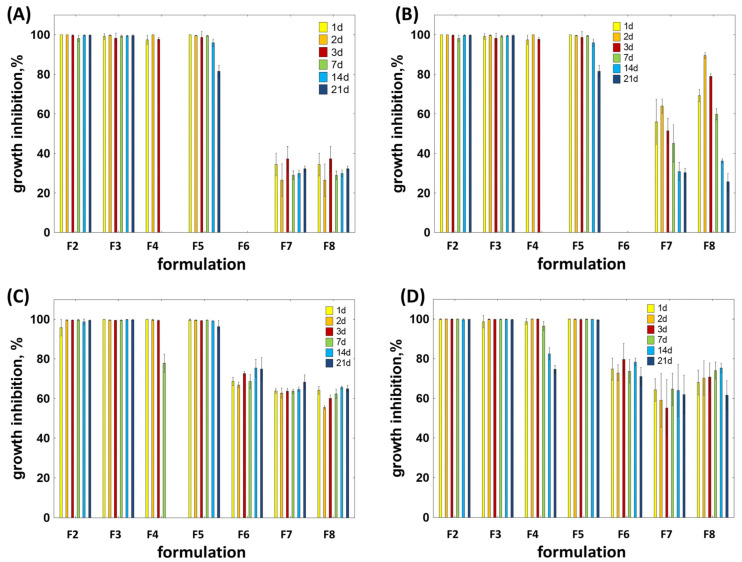
Percentage of microbial growth inhibition of active substances released from implants to PBS after 1, 2, 3, 7, 14, and 21 days of immersion, tested against (**A**) *E. coli*, (**B**) *S. aureus*, (**C**) *S. mutans*, (**D**) *S. sobrinus* (n = 3).

The antimicrobial activity of the substances released from the implants was evaluated using 96-well plates, and the results are presented in Figure 5.

For *E. coli* (Figure 5A), complete (100%) bacterial growth inhibition from day 1 to day 21 was observed only in the presence of DOXY (F2) and DOXY + EUG (F3). Minocycline released from formulation F4 fully inhibited the growth of this strain only up to day 3, whereas the addition of eugenol in formulation F5 prolonged its effective antimicrobial activity to day 7. While F4 showed no inhibition at later time points, formulation F5 maintained an 85% growth inhibition on day 21. Solutions containing only EUG released from F6 did not affect bacterial growth, whereas solutions released from implants F7 and F8, containing 30% and 50% EUG, respectively, reduced bacterial growth by approximately 30%. From day 1 to day 21, solutions from formulations F2, F3, and F5 achieved complete (100%) inhibition of *S. aureus* growth (Figure 5B). MIN alone (F4) remained effective up to day 7. In contrast, EUG alone (F6 to F8) exhibited a moderate inhibitory effect, reducing bacterial growth by 42–90%. Both DOXY alone and in combination with EUG completely inhibited the growth of *S. mutans* (Figure 5C) and *S. sobrinus* (Figure 5D). MIN released from formulation F4 reached effective inhibitory concentrations for *S. mutans* only up to day 3, whereas for *S. sobrinus,* up to day 7. The addition of EUG in formulation F5 extended the antimicrobial activity. However, EUG alone (F6–F8) moderately inhibited the growth of both strains, with inhibition ranging from 55% to 80%.

As no differences were detected between formulations F2 and F3 in the microplate assays, both of which were fully effective, additional experiments were conducted using the agar well diffusion test to illustrate the effect of EUG further. Wells were filled with solutions containing the substances released from the formulations, allowing the active agents to diffuse slowly through the agar. Results are presented in Table 3 and Figure S3 (Supplementary Information).

The smallest inhibition zones were observed for *E. coli*. For formulation F2 (DOXY), the mean inhibition zone after 1 day was 18.7 mm, while no inhibitory effect was detected on day 21. In contrast, for the Gram-Positive strains, the inhibition zones exceeded an average of 31 mm on day 1, whereas on day 21, an inhibitory effect was recorded only for *S. aureus*, with a mean inhibition zone of 13 mm. Notably, a statistically significant difference was observed between solutions F2 and F3. The addition of EUG (F3) not only increased the inhibition zones, from 22.3 mm for *E. coli* to 40 mm for *S. sobrinus*, but also prolonged the antibacterial effect up to day 21 of release.

Both *Streptococcus* strains are known for their strong biofilm-forming ability, which significantly hinders their removal from diseased sites [44]. Therefore, the biofilm-forming ability of the microorganisms was evaluated by SEM observations in the presence of EUG released from formulations F6–F8, as well as from F3, which was identified as the most promising formulation in terms of physicochemical and antibacterial properties among those loaded with the antibiotics.

As shown in Figure S4, both strains efficiently colonized polypropylene surfaces incubated in both BHI medium, serving as a positive control, and the EUG solution released from formulation F6, with the lowest EUG concentration. In contrast, the higher EUG concentrations allowed only small colonies (F7) or single cells (F8) of S. mutans to form, while S. sobrinus did not grow. Additionally, the mixture of DOXY and EUG released from the F3 formulation effectively prevented the growth of both strains.

**Figure 6:**
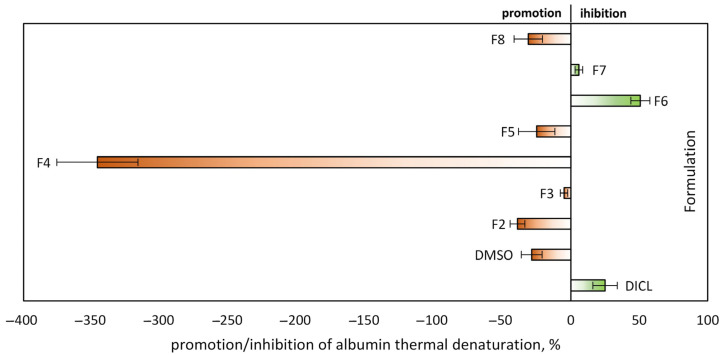
Percentage of promotion/inhibition of protein thermal denaturation of ISFI formulations after 1 day of the release experiment; diclofenac sodium (DICL)—positive control; DMSO—negative control (n = 3).

Because periodontitis is strongly linked to inflammation, the present study, in addition to assessing antimicrobial efficacy, also focused on evaluating the pro- and anti-inflammatory potential of the tested formulations [45]. The active substances released from the individual formulations on the first day were also assessed for pro- or anti-inflammatory activity using the albumin thermal denaturation inhibition assay. As shown in Figure 6, sodium diclofenac (positive control) and formulations F6 and F7, containing EUG as the sole active substance, exhibited protective effects on proteins against thermal denaturation. Notably, formulation F6, with the lowest EUG concentration, provided the strongest protective effect, whereas increasing the EUG concentration reduced the anti-inflammatory activity, with formulation F8 even inducing a pro-inflammatory (pro-denaturing) response. The assay further demonstrated that combining EUG with DOXY or MIN (formulations F3 or F5, respectively) significantly enhanced protein protection compared with the antibiotics alone.

## 3. Discussion

The treatment of periodontitis presents a considerable challenge, as conventional drug administration routes (such as oral, intravenous, subcutaneous) require high systemic drug levels, while drug concentration at the target site remains low. Additionally, the rapid flow of gingival crevicular fluid accelerates the clearance of drugs from the site of action. In contrast, injectable ISFI systems can be easily placed into the periodontal pocket and provide controlled drug release over a prolonged period. An additional advantage of ISFI systems is that the geometry and size of the implant can be easily adapted to the individual patient’s periodontal pocket [46]. The stability and durability of the implant within the periodontal pocket mainly depend on its flexibility and susceptibility to hydrolytic degradation, which are determined by the type and properties of the polymer.

In our studies, PISEB was proposed as the polymeric component of novel ISFI formulations designed for the controlled and prolonged delivery of active agents to sites of acute or chronic bacterial infection within periodontal tissues. The suitability of PISEB for biomedical applications, owing to its lack of toxicity, was verified in our previous work with human umbilical vein endothelial cells (HUVEC) [21]. Based on the MTT assay, HUVEC viability in contact with the PISEB surface was 91 ± 3%, only slightly lower than that observed for the tissue culture plastic control (100%). Another advantage of PISEB in ISFI systems is its relatively low glass transition temperature (~2 °C). This implies that under physiological conditions (37 °C), PISEB exists in a highly elastic state, which may facilitate adaptation of the implant to the periodontal pocket cavity. In contrast, the polymers currently used in ISFI systems, PLA and PLGA, exhibit considerably higher glass transition temperatures, ranging from 57 to 65 °C and 45 to 55 °C, respectively [13]. If the glass transition temperature (Tg) of the polymer is above body temperature, the polymer remains in a “frozen” glassy state, which significantly limits molecular mobility and results in a rigid, brittle material. The hydrolysis of PISEB under conditions similar to the physiological environment proceeds relatively slowly, as reported by Park et al. [47], and leads to the release of IS and sebacic acid (SBA). SBA can be fully metabolized to acetyl-CoA, which enters the Krebs cycle, thereby confirming its safety for biomedical applications. IS, in turn, has been verified as safe and non-toxic in ADME studies conducted in rats [22,48]. The antibiotics DOXY and MIN were selected as they are already employed in commercial systems for local periodontal therapy [5,6]. EUG was incorporated as a natural antibacterial and anti-inflammatory additive, intended to enhance the antibacterial activity of antibiotics against periodontal pathogens.

Our results demonstrated that the particles of both antibiotics were insoluble in liquid PISEB-based formulations. Consequently, they were drug suspensions within the polymer solution. All formulations displayed shear-thinning behavior except the basic formulation F1 (without antibiotic or EUG), which was characterized by constant viscosity across the entire range of shear rates. It is known that the rheological behavior of dilute suspensions of non-spherical, rigid particles is shear-thinning non-Newtonian [49]. Under no flow condition, the non-spherical particles are randomly oriented in the matrix fluid. At a high shear rate, the particles become aligned or oriented with the flow, resulting in reduced viscosity of the suspension [50]. The shear-thinning behavior may be advantageous for injectable ISFI drug delivery systems. During injection, the applied shear force lowers the viscosity, allowing the formulation to pass through standard needles. After injection, upon removal of the shear force, the formulation becomes more viscous. All PISEB-based ISFI formulations could be injected through a 21-gauge syringe needle (inner diameter, 0.60 mm; length, 40 mm), regardless of their flow behavior. The selected needle size is comparable to other ISFI products available commercially (23 G for Atridox^®^, 20 G needle for Eligard^®^) [8].

The morphology of the depot formed after injection and phase inversion is influenced by the composition of the ISFI liquid formulation. According to reports on PLGA-based ISFI systems, the key factor is the solvent and its affinity for water [51]. Hydrophobic solvents (e.g., triacetin, benzyl benzoate) undergo phase inversion slowly. They diffuse from the depot at a reduced rate, which slows down water penetration, and the resulting implant structure can be described as uniformly dense with a limited number of pores [52]. In contrast, hydrophilic solvents with high water affinity, such as NMP or DMSO, undergo rapid phase inversion and tend to form porous implants. This is due to the rapid migration of the solvent from the formulation into the surrounding tissue, accompanied by water penetration into the polymer solution. As a result, the formation of a thin membrane with a highly porous implant structure is observed. DMI is a polar solvent that, like DMSO, is fully miscible with water at any ratio [53]. Therefore, rapid polymer precipitation occurs, resulting in the formation of highly porous depots. The addition of EUG, which is more hydrophobic, resulted in the formation of depots with a less porous and denser morphology. Our SEM observations of the microstructure of precipitated PISEB-based depots are in good agreement with the morphology of PLGA-based implants reported by other authors [54,55,56,57].

In ISFI systems, the drug release profile and the initial burst release are influenced by the dynamics of phase inversion and the rate of implant formation, both of which depend on the water miscibility of the solvent. The burst release, defined as the percentage of drug released during the first 24 h after injection, is typically high [8,52]. In fast-inverting systems such as ISFI based on water-miscible NMP, the burst release is relatively high. By introducing a hydrophobic cosolvent, this effect can be reduced [51].

In our studies, the hydrophilic solvent DMI was used together with EUG, which is more hydrophobic, to modify the release behavior of the antibiotics. The burst release of both antibiotics was relatively high (ranging from 40% to 86%), indicating that ISFI systems with DMI as the solvent should include a hydrophobic cosolvent to achieve prolonged release with a more moderate initial burst. The addition of EUG (10 wt.% of DMI) extended the release of DOXY and MIN, while reducing the burst release of MIN. A similar effect of clove oil on DOXY release from ISFI systems based on Eudragit RS and NMP was previously reported by Phaechamud [58]. It is possible that eugenol’s chemical structure may influence the release of drugs such as doxycycline or minocycline hydrochloride by forming non-covalent interactions—including hydrogen bonding and hydrophobic interactions—that could lead to the formation of transient complexes between eugenol and these antibiotics. Such interactions may potentially affect their solubility and diffusion from the polymer matrix. Moreover, because eugenol is lipophilic, it may also modify the polymer network itself, altering its porosity and overall release profile. However, no data on these effects are currently available in the literature, and experimental confirmation is required.

The observed differences in burst release values of the investigated antibiotics can be attributed to their solubility in PBS medium at 37 °C, which is reported as 1.85 mg/mL for DOXY and 6.79 mg/mL for MIN [59,60]. The more porous surface morphology of the F4 depot (containing MIN alone), compared with that of F2 (containing DOXY alone), further supports this explanation, as MIN can be readily dissolved and removed from the implant surface due to its higher solubility, whereas such an effect is not observed for DOXY with its considerably lower solubility.

The release profile of DOXY and MIN fits well with the Higuchi kinetic model. Higuchi’s model describes the controlled release of an active substance from a polymer matrix through diffusion, assuming an insoluble (or slowly soluble) matrix, negligible (or very slow) matrix degradation, and a release process primarily controlled by diffusion through the polymer [61]. Since PISEB is hydrophobic and does not degrade during the 21-day experiment, it perfectly meets the model’s assumptions. Interestingly, the release profile of EUG was independent of its content in the formulation and followed the same pattern in all cases, consistent with a first-order kinetic model. It means that the amount of EUG released is proportional to the amount of remaining EUG in implants. Thus, the amount of active agent released tends to decrease with time.

In the subsequent stage, biological assays were conducted to verify whether concentrations of the released active compounds remained within the therapeutic range, rather than sub-therapeutic ones that could promote the selection of resistant strains, to evaluate their ability to inhibit bacterial biofilm formation, and to examine potential interactions between the formulation components, which could either enhance or diminish the antimicrobial and anti-inflammatory effects of the active agents.

Due to the rapid development of technologies for identifying microorganisms inhabiting the human body, the amount of information regarding the composition of healthy oral microbiota has increased significantly in recent years. Studies have shown that in diseases affecting teeth and the periodontium, the proportion of beneficial bacteria decreases. These are often replaced by other microorganisms, which may not be typical for the oral cavity, depending on the severity of the inflammatory process and the disease [62].

In our study, *E*. *coli* and *S*. *aureus* were selected as reference strains to evaluate the antimicrobial activity of the new formulations. Nevertheless, *E. coli* is also frequently isolated from periodontal pockets, predominantly from patients with moderate and chronic periodontitis [63,64]. Similarly, *S. aureus* has been detected in subgingival samples of periodontitis patients, indicating its relevant role in periodontal dysbiosis [65]. Additionally, we used two other strains: *S. mutans* and *S. sobrinus*, which, although classically associated with dental caries, have also been shown to play a role in periodontal and endo-periodontal lesions [66]. Their strong biofilm-forming capacity and the production of collagen-binding proteins facilitate adhesion to root surfaces, thereby contributing to the progression of periodontal disease [67,68]. Moreover, it has been shown that *S. sobrinus* supports other periodontopathogens, enhancing their pathogenic potential [69].

The first compound tested within the formulations was EUG, whose antibacterial activity is primarily associated with the disruption of bacterial membranes and cell walls, leading to the leakage of intracellular contents and cell death [70]. It was observed that solutions containing solely EUG, released from formulations F6 to F8, caused only partial inhibition of microbial growth. Interestingly, the inhibitory effect was largely independent of the EUG concentration in the samples. In general, the strongest growth suppression was observed for *S. aureus*, the weakest for *E. coli*, and an intermediate effect for streptococci. The low sensitivity of *E. coli* to EUG was attributed to the presence of an additional outer membrane that hinders penetration of active compounds into the cells [71]. For all tested strains, the inhibitory effect did not significantly depend on EUG concentration in the solutions. This was most likely because the final EUG concentration, after addition of bacterial suspensions released from the formulations, did not reach the MIC values previously determined for *E. coli* (1250 µg mL^−1^) and *S. aureus* (625 µg mL^−1^). It is noteworthy that these MIC values were considerably higher than those reported in the literature [42,72]. For both strains, at any concentration below the MIC, we observed constant microbial proliferation monitored between 20 and 24 h of incubation. A different pattern was observed for *S. mutans* and *S. sobrinus*, for which the determined MIC values (1250 µg mL^−1^) after 24 h of incubation with EUG also exceeded previously reported literature values [44,73]. This was due to the fact that only at this concentration did EUG inhibit bacterial growth entirely from the start of incubation. Comparing the results of microbial growth inhibition at 24 and 30 h, complete suppression of both streptococcal strains was observed, even at an EUG concentration as low as 0.0095 µg mL^−1^. This phenomenon aligns with the literature, which indicates that the detected antibacterial efficacy of EUG is time-dependent. During the time-kill test, the strains proliferated for the first few hours, after which bacterial growth inhibition was observed [44]. Therefore, the observed lack of complete growth inhibition for the streptococcal strains can be attributed solely to their capacity to multiply in the formulation solutions during the initial incubation period.

The effects of EUG released from formulations F6–F8 on *S. mutans* and *S. sobrinus*, both known for their biofilm-forming capacity, were also evaluated by SEM (Figure S4). The results demonstrated that, in contrast to inhibiting planktonic cell proliferation, EUG suppressed biofilm formation in a concentration-dependent manner. The EUG concentration released from formulation F6 after 24 h was insufficient to prevent bacterial adhesion and subsequent biofilm formation. In contrast, increasing EUG concentrations markedly reduced the number of microbial cells colonizing the polypropylene surface. This effect may be attributed to the reported ability of EUG to interfere with biofilm formation and development [42]. Since the establishment of a mature biofilm reduces bacterial susceptibility to antibiotics, it is crucial to block the initial adhesion of bacteria to surfaces, thereby preventing the formation of microcolonies that constitute the foundation of biofilm architecture [74]. There are, however, existing studies that indicate that combining eugenol with tetracyclines (or other natural essential oils and antibiotics) can also eradicate already established bacterial biofilms. Miladi et al. [75] demonstrated that EUG, either alone or in combination with tetracycline, was capable of penetrating the polysaccharide matrix of the biofilm and disrupting its structural integrity.

Since the tested formulations are intended for periodontitis treatment, it is essential that, in addition to their antibacterial activity, they also exhibit anti-inflammatory properties, which accelerate tissue healing and hinder colonization by pathogenic microorganisms. In our study, the protective effect of eugenol on albumin proteins was shown to be concentration-dependent. EUG concentrations ranging from 0.63 mM (F6) to 2.69 mM (F7) prevented thermal protein denaturation, whereas at 4.0 mM (F8), the opposite, pro-denaturing effect was observed. Literature reports indicate that the pro-oxidative effect of EUG may contribute to its cytotoxicity by destabilizing protein structures through binding to lysine residues, thereby promoting denaturation [76]. Such a biphasic, dose-dependent phenomenon, known as hormesis, has been described in multiple reports [77]. For example, Koh et al. [78] demonstrated that low concentrations of eugenol (5 µM) stimulated interleukin-8 secretion in human pulp cells (HPC), indicating an anti-inflammatory effect. In contrast, higher concentrations (0.5 mM) elicited a pro-inflammatory response. Moreover, exposure of oral human cells to EUG for more than 4 h induced irreversible, non-apoptotic cell death [78].

In the microdilution assays with solutions released from formulations F2 (DOXY) and F3 (DOXY with EUG), complete growth inhibition (100%) of all tested bacterial strains was observed throughout the 21-day experimental period. In all tested release media, the concentration of DOXY exceeded the determined MIC values, which were 2.0 µg mL^−1^ for *E. coli*, 1.0 µg mL^−1^ for both *Streptococcus* strains, and 0.5 µg mL^−1^ for *S. aureus*. Differences in antibacterial activity between formulations F2 and F3 were, however, revealed by well agar diffusion assays, where an apparent adjuvant effect of eugenol was observed. These tests showed that the presence of eugenol in formulation F3 not only enhanced but also prolonged the antibacterial effect of DOXY released from the implants. Moreover, SEM micrographs demonstrated that in the presence of DOXY combined with EUG (F3), both *Streptococcus* strains were unable to adhere to solid surfaces, in contrast to formulation F6, which contained the same amount of EUG (10%) as the sole active agent.

Microdilution studies demonstrated that solutions obtained from both implants containing MIN inhibited the growth of all bacteria by 100% only during the first three days of the experiment. This effect was most likely due to the rapid burst release of MIN from both formulations (F4 and F5) within the first day. Since all tested bacterial strains were susceptible to minocycline (MIC value 0.6 µg mL^−1^), the residual drug released over the following two days effectively suppressed bacterial proliferation. Beyond this period, incomplete bacterial inhibition was observed. Clinically, this is a highly unfavorable phenomenon, as the emergence of strains with reduced antibiotic susceptibility develops under the constant selective pressure of sublethal antibiotic doses, as previously demonstrated for dental plaque bacteria resistant to MIN [79]. Importantly, in the case of the formulation containing both MIN and EUG (F5), the antibacterial effect of the released solutions was stronger and extended over a longer period. On day 21 of the experiment, 100% growth inhibition was observed for the Gram-Positive strains, and 85% inhibition was recorded for *E. coli*.

The higher susceptibility of all tested strains to DOXY and MIN in the presence of EUG (formulations F3 and F5) can be explained by at least two mechanisms. First, the addition of EUG slowed down the release of antibiotics from the system (as described earlier), ensuring that the drug concentration remained above the MIC values throughout the experiment. Second, plant-derived compounds such as eugenol are known to act directly on microbial cells by disrupting cell membranes and interfering with selective transport across the cell wall, which may facilitate antibiotic penetration. For example, Moon et al. [44] demonstrated that combining eugenol with ampicillin or gentamicin increased the sensitivity of periodontal pathogens by 4- to 8-fold compared to the antibiotics alone. Similarly, Valcourt et al. [80] reported that the combination of doxycycline with essential oils enhanced the susceptibility of Gram-negative strains, including *E. coli* ATCC 25922, to antibiotics. Interestingly, in their study, the addition of essential oils did not inhibit bacterial growth but exerted a bactericidal effect. Existing studies do indicate that combining eugenol with tetracyclines (or other natural essential oils and antibiotics) can enhance antibacterial activity or modulate resistance.

The albumin thermal denaturation inhibition assay performed with solutions released from antibiotic-loaded formulations revealed a pro-denaturing effect for all tested systems. This effect was particularly pronounced for formulation F4, which contained MIN alone, where the strongest promotion of protein denaturation was observed after the first 24 h of release. In our study, after the first day of the experiment, the MIN concentration reached approximately 1 mg mL^−1^, which could have been the direct cause of protein inactivation. By comparison, in the commercially available formulation Periocline^®^, the initial burst release of minocycline reaches approximately 1300 µg/mL within the first 7 h [6]. The promotion of protein denaturation under certain conditions has already been reported for tetracyclines, where antibiotics bind serum albumin and alter its conformation and thermal stability [81]. It is also noteworthy that the protein-denaturing effect observed in the presence of MIN in our experiments was about ten times stronger than that induced by DMSO, which served as a negative control at a concentration of 15% *v*/*v*. According to the literature, the impact of DMSO on protein solutions is also concentration-dependent, with decreases in protein thermodynamic stability (lowering of Tm) being observed at concentrations ranging from 5% to 20%, depending on the protein tested [82].

These experiments demonstrated that the high concentration of MIN released after the first day caused rapid protein denaturation, simulating a pro-inflammatory effect. Several earlier studies have indicated that minocycline, although widely recognized as one of the best-documented antibiotics with anti-inflammatory and neuroprotective activity [35], may exert the opposite effect when administered at excessively high concentrations. For example, Liu et al. [83] demonstrated that minocycline at 20 and 40 µg mL^−1^ was toxic to neural progenitor cells (NPCs). Other studies [84] investigating the effects of minocycline at concentrations exceeding 200 µg/mL on oral epithelial cells reported necrotic and apoptotic changes in over 50% of the cells. These findings suggest that prolonged exposure to such concentrations may contribute to clinical local irritation and potentially disrupt periodontal tissues. Although actual tissue levels in periodontal pockets may differ from in vitro observations [85], controlled slow release of minocycline may mitigate pro-inflammatory effects, maintain concentrations above the MIC of target pathogens, and help reduce the risk of resistant strains. Slowing down drug release and reducing burst release can be achieved by modifying the composition of the formulation. This modification may involve the use of a polymer with a higher molecular weight or a more hydrophobic cosolvent [52]. The simplest method of modification would be to increase the concentration of the polymer, which would result in a thicker polymer skin that would slow down the solvent-water exchange and the associated diffusion of the drug [51]. However, this may cause a significant increase in the viscosity of such a system, making it difficult to inject into the gingival pockets.

Our studies demonstrated the anti-inflammatory effect of eugenol at concentrations released from formulations F6 and F7. However, eugenol released from formulation F8, which had the highest content of this compound, had a pro-inflammatory effect. The eugenol content in all tested formulations was higher than in typical topical dental applications, ranging from 0.1% to 2% for short-term rinses and up to 5% in controlled-release formulations, such as mucoadhesive gels or nanoparticles. In our studies, the use of higher-than-typical concentrations of eugenol, based on the previously determined MIC values, completely prevented the growth of microorganisms from the very beginning of the experiment. Indeed, cytotoxicity has been observed in vitro at concentrations of EUG exceeding 5% in gingival and oral mucosal fibroblasts [86,87]. It should be noted, however, that these in vitro findings may not directly translate to in vivo conditions, as local concentrations in gingival crevicular fluid or tissues have not been quantified in humans.

In our work, eugenol at anti-inflammatory concentrations (F6) substantially reduced the pro-inflammatory effect of both antibiotics on day 1 of release. The response decreased eightfold for DOXY and fourteenfold for MIN. This finding indicates an additional positive effect of eugenol. A similar observation was reported by Said [88], who demonstrated in a rat model that co-administration of eugenol with gentamicin mitigated nephrotoxicity and inflammatory damage.

## 4. Materials and Methods

### 4.1. Synthesis of Poly(Isosorbide Sebacate) (PISEB)

Isosorbide (IS, 98%) (Acros Organics, 10153491) was used to synthesize after drying under vacuum at 40 °C. Diethyl sebacate (98%) was purchased from Sigma-Aldrich, St. Louis, MO, USA (W237604) and used as received. Novozyme^®^ 435 (lipase B from *Candida antarctica* immobilized on crosslinked polyacrylate beads) was purchased from STREM Chemicals Inc., Newburyport, MA, USA (06-3123-25G).

Poly(isosorbide sebacate) (PISEB) was synthesized using the enzymatic polytransesterification method as previously described [20,21,89]. Synthesis proceeded as follows: isosorbide (1.46 g, 0.01 mol) and diethyl sebacate (2.58 g, 0.01 mol) were placed in a 100 mL round-bottomed flask with a molar ratio of 1:1 under a dry argon atmosphere. Reagents were heated in a solvent mixture (cyclohexane/benzene = 6:1 *v*/*v*, 50 mL) in the presence of Novozyme^®^ 435 (10 wt.% of the monomers), used as a polyesterification catalyst. The reaction mixture was refluxed with the aid of a Dean-Stark apparatus for 7 days, under a dry argon atmosphere. During the reaction, a Dean-Stark attachment was filled with 4 Å molecular sieves to remove ethyl alcohol, a by-product of the reaction, from the reaction mixture. The molecular sieves were replaced every 24 h. The obtained polyester was dissolved in chloroform to separate it from the catalyst, and then the polymer solution was slightly concentrated using a rotary evaporator. Polyester was precipitated in methanol, filtered, and dried under vacuum to a constant mass (1 mmHg, 40 °C, 24 h). The expected structure of PISEB was confirmed by ^1^H NMR and ^13^C NMR, and its thermal properties were analyzed by DSC. NMR spectra of PISEB and its DSC thermograms are presented in Figures S5 and S6 (Supplementary Information). Number-average (Mn), weight-average (Mw) molecular weight, and dispersity (D) of PISEB were determined by GPC: Mn = 14,600, Mw = 26,200, D = 1.79.

PISEB ^1^H NMR (CDCl_3_, ppm) δ: 5.20–5.15 (s, 1H, C**H**OC(O) IS, exo), 5.10–5.15 (m, 1H, C**H**OC(O) IS, endo), 4.80–4.85 (m, 1H, -C**H** IS), 4.45–4.50 (d, 1H, -C**H** IS), 3.90–4.00 (m, 3H, -CH_2_ IS), 3.75–3.80 (m, 1H, -CH_2_ IS), 2.35–2.40 (t, 2H, -OC(O)-C**H**_2_CH_2_-, sebacic, exo), 2.30–2.35 (t, 2H, -OC(O)-C**H**_2_CH_2_-, sebacic, endo), 1.55–1.65 (m, 4H, -OC(O)-CH_2_C**H**_2_-, sebacic), 1.20–1.35 (m, 8H, -CH_2_(C**H**_2_)_4_-CH_2_-, sebacic).

PISEB ^13^C NMR (CDCl_3_, ppm) δ: 173.1 (C=O, exo), 172.8 (C=O, endo), 85.9 (-CH IS), 80.7 (-CH IS), 77.8 (-**C**HOC(O) IS), 73.7 (-**C**HOC(O) IS), 73.4 (-CH_2_ IS), 70.3 (-CH_2_ IS), 34.1 (-OC(O)-**C**H_2_CH_2_-, sebacic, exo), 33.9 (-OC(O)-**C**H_2_CH_2_-, sebacic, endo), 28.9 (OC(O)-CH_2_**C**H_2_-, sebacic), 24.7 (-CH_2_(**C**H_2_)_4_-CH_2_-, sebacic). DSC: Tg = 1.49 °C, Tm = 54.76 °C (1st heating run), Tg = 2.93 °C (2nd heating run).

### 4.2. Preparation of PISEB-Based Formulation

Dimethyl isosorbide (DMI, 99%) (Acros Organics, 10272762) and other organic solvents were purchased from Fisher Scientific, Hampton, NH, USA (and used without purification. The drugs used in this study were two tetracyclines. First, doxycycline hyclate (DOXY, >98%, Sigma-Aldrich, D9891) and the second, minocycline hydrochloride (MIN, 99%, Angene, Secunderabad, India, AGN-PC-0WGD4D), which are present as yellow hygroscopic crystalline powders. Eugenol (EUG, 99%) (Acros Organics, 10607652) was used as received. Phosphate-buffer solution (PBS) (NaH_2_PO_4_/Na_2_HPO_4_) with a pH of 7.41 was obtained from Gibco (10010023).

Homogeneous polymer solutions were prepared by dissolving solid polyester into dimethyl isosorbide (DMI) over 24 h at ambient temperature. The liquid PISEB formulations containing minocycline hydrochloride (MIN) or doxycycline hyclate (DOXY) were prepared by the addition of drug powder (200 mg, 10% *w*/*w*, based on the total liquid formulation) to the solution containing 400 mg PISEB in 1600 mg of solvent. PISEB-based liquid formulation was mixed with MIN or DOXY at room temperature until a uniform mixture was achieved. Firstly, the mixtures were combined using a spatula, and then they were unified for a minute using the mini laboratory shaker LabDancer. The liquid PISEB formulations containing eugenol (EUG) were prepared by replacing an appropriate amount of the solvent DMI with EUG. The freshly prepared mixtures (100 µL) were injected immediately into PBS buffer (pH 7.4, 10 mL, temp. 37 °C) in a glass vial (ϕ = 12 mm) through a 21-gauge needle of 40 mm length, using a disposable syringe (1 mL) and set aside in the laboratory incubator at 37 °C for a required period of time.

### 4.3. Rheology

To determine the viscosity-versus-shear rate dependence of PISEB-based formulations, flow experiments were run in a continuous ramp mode using a Discovery HR20 Rheometer (TA Instruments, Antwerp, Belgium). During the tests, a stainless steel cone plate with a cone angle of 1° was used. The radius of the plates and the gap between them were 20 mm and 80 mm, respectively. Measurements were performed in triplicate at room temperature (25 °C).

### 4.4. In Vitro Drug Release Study

The antibiotics and EUG release experiments were conducted by injecting the formulation (100 μL) into a vial containing 10 mL of PBS (pH 7.4) at 37 °C, followed by incubation at the same temperature for 21 days. At each time point, the medium was collected and replaced with 10 mL of fresh PBS, a process repeated up to 21 days. The drug concentration in the collected PBS solutions was analyzed spectrophotometrically (HITACHI U-2910 spectrophotometer, Tokyo, Japan) at λmax of 352 nm, 325 nm, and 279 nm for MIN, DOXY, and EUG, respectively. The proper wavelength was selected based on studies conducted on the stability of MIN and DOXY in PBS. Each experiment was performed in triplicate, and the results are presented as mean values ± standard deviation.

### 4.5. Analysis of the in Vitro Release Kinetics

The data obtained from the in vitro release of DOXY, MIN, and EUG from different formulations were fitted by different mathematical kinetic models. The best kinetic model to fit active agent release data from different implants was selected based on the r^2^ criterion, which demonstrates the correlation between the model and experimental data. The following kinetic models were used to evaluate the in vitro drug release: (1) zero-order model (cumulative drug release (%) vs. time, Equation (1); (2) first-order model (log cumulative of drug remaining (%) vs. time, Equation (2); (3) Higuchi model (cumulative drug release (%) vs. square root of time, Equation (3).

Q_t_ = Q_0_ + k_0_t (1)

logQ_t_ = logQ_0_ − k_1_t/2.303 (2)

Q_t_ = k_H_t^1/2^ (3)

where Q_t_ is the amount of drug released at time t, Q_0_ is the initial amount of drug in solution (equal to zero), k_0_ is the zero-order release constant (concentration·time^−1^), k_1_ is the first-order release constant (time^−1^), k_H_ is the Higuchi dissolution constant (time^−1/2^).

### 4.6. Characterization of PISEB and Precipitated Depots

The molecular weight of PISEB was determined by gel permeation chromatography (GPC) using an Agilent Technologies chromatograph equipped with a differential refractometer detector (MDS RI Detector, Agilent Technologies, Santa Clara, CA, USA) and calibrated with polystyrene standards. The measurements were carried out in methylene chloride (HPLC grade) as the solvent at 30 °C with a flow rate of 0.8 mL min^−1^. The structure of PISEB was analyzed using ^1^H and ^13^C NMR Spectroscopy in CDCl_3_ on a 600 MHz spectrometer (Varian, Palo Alto, CA, USA).

Differential scanning calorimetry (DSC) was performed on a METTLER TOLEDO DSC3 differential scanning calorimeter (METTLER TOLEDO, Columbus, OH, USA) at a heating rate of 10 K min^−1^ under an inert gas. The DSC thermograms were recorded for both the first and second heating runs, spanning a temperature range of −50 to 100 °C. The melting temperature (Tm) and glass transition temperature (Tg) were measured at the first and the second heating scans, respectively.

The internal and surface morphology of the depots formed after injection into the aqueous phase was studied using a scanning electron microscope (SEM, Phenom ProX, Thermo Fisher Scientific, Waltham, MA, USA), operating at an accelerating voltage of 10 kV. After 1 day of incubation, the samples were washed carefully with distilled water and freeze-dried for 24 h. Afterward, all samples were affixed to a metal stub using carbon double-sided adhesive and sputter-coated with a thin gold layer (5 nm) before observation (20 min, 20 mA; Q150R Quorum Technologies, Laughton, UK).

### 4.7. In Vitro Antibacterial Activity Study

#### 4.7.1. Microorganisms

The four model microorganisms, *Escherichia coli* ATCC 25922, *Staphylococcus aureus* ATCC 25923, *Streptococcus mutans* ATCC 25175, and *Streptococcus sobrinus* ATCC 33478, selected for this study, were obtained from the American Type Culture Collection (ATCC). *E. coli* and *S. aureus* were used as reference strains, while *S. mutans* and *S. sobrinus* are known oral pathogens [90]. Before each experiment, bacteria were thawed (from –80 °C) and transferred to the appropriate media: Mueller–Hinton broth (MHB) (Oxoid, CM0405B) supplemented with divalent cations for *E. coli* and *S. aureus*, or Bacto Brain Heart Infusion (BHI) (BD, 237500) for *S. mutans* and *S. sobrinus*. Cultures were incubated at 37 °C with shaking at 130 rpm for 24 h. In parallel, the purity of the strains was monitored on selected agar-solidified media (BTL, S-0030) to exclude potential contamination.

#### 4.7.2. Minimum Inhibitory Concentration Determination

The broth microdilution method was applied to confirm the susceptibility of the selected strains to the tested substances [91]. The minimum inhibitory concentration (MIC) for each strain was determined using serial twofold dilutions of (1) antibiotics in the range of 128–0.125 µg mL^−1^ and (2) eugenol in the range of 20–0.0095 mg mL^−1^, dispensed into the wells of microtiter plates. Bacterial inocula were prepared as described in [92]. Briefly, 24 h bacterial cultures were subcultured into fresh medium and incubated at 37 °C with shaking (130 rpm) for 2–6 h, until an optical density (OD600) of approximately 0.5 was reached. The cultures were then diluted to obtain bacterial suspensions at a density of 1 × 10^6^ CFU (colony-forming units). Within 30 min of preparation, 100 µL of bacterial suspension was distributed into wells containing 100 µL of antibiotic or eugenol solution. For each strain, experiments were performed in six replicates. Plates were incubated (Binder incubators: BD 240 or CB53, CO_2_ incubator) at 37 °C for 20–24 h, after which OD600 was measured using a SparkTM Multimode Microplate Reader (Tecan Group Ltd., Männedorf, Switzerland).

#### 4.7.3. Antibacterial Activity

The antibacterial activity of F2–F8 solutions released from implants during the 21-day study was evaluated using the broth microdilution method, as described in Section 4.7.2. Within 30 min of inoculum preparation, 100 µL of bacterial suspension was distributed into wells of a microtiter plate containing 100 µL of F2–F8 solutions. For each bacterial strain, experiments were performed in three replicates. Plates were incubated at 37 °C for 20–24 h, and the optical density at 600 nm (OD600, hereafter referred to as OD_BF_) was measured using a SparkTM Multimode Microplate Reader (Tecan Group Ltd., Männedorf, Switzerland). Non-inoculated broth served as the first negative control (OD_M_), and F2–F8 solutions mixed with broth medium (1:1) served as the second negative control (OD_F_). Media inoculated solely with bacteria served as the positive control (OD_B_). The percentage of bacterial growth inhibition was calculated using the following formula:

(4) $$\%GI=\frac{{(OD}_{B}-{OD}_{M})-({OD}_{BF}-{OD}_{F})}{{(OD}_{B}-{OD}_{M})}\times 100\%$$

where

GI—growth inhibition;

OD_B_—OD600 of positive control;

OD_M_—OD600 of negative control (non-inoculated medium);

OD_BF_—OD600 of bacteria incubated with F2 to F8 solutions released from implants;

OD_F_—OD600 of negative control (medium mixed with released solutions (F2 to F8)).

For the visualization of differences in antimicrobial activity among promising formulations, the selected solutions were further tested using the agar well diffusion method. Briefly, 100 µL of each bacterial inoculum (1 × 10^6^ CFU) was spread onto the surface of the appropriate agar medium (see Section 4.7.1). After drying, 8 mm wells were cut in the agar and filled with 50 µL of F2 and F3 solutions. Plates were incubated at 37 °C for 20–24 h, after which zones of inhibition were recorded.

#### 4.7.4. Biofilm Formation

To assess whether the concentration of eugenol released from F3, F6, F7, and F8 implants affects the ability of *S. mutans* and *S. sobrinus* to form biofilm within 24 h, bacterial suspension (1 × 10^6^ CFU) prepared as described in Section 1 was mixed in a 1:1 ratio with the tested solutions in 12-well plates containing sterile PCR tube lids at 37 °C for 24 h. Bacterial cultures incubated in BHI medium served as positive controls. After incubation, lids were mounted on SEM sample holders, dried in a desiccator for 24 h, and sputter-coated with a 10 nm gold layer (Q150R Quorum Technologies, Laughton, UK). SEM observations were performed as described in Section 4.6.

### 4.8. Anti-Inflammatory Test

The ability of F2–F8 solutions to inhibit thermal denaturation of albumin was evaluated using a modified method [93]. Briefly, 1.2 mL of F2–F8 solutions released from implants after the first day of the experiment was mixed with 1.2 mL of PBS, which served as the negative control, and the absorbance at 660 nm was measured using a spectrophotometer (Shimadzu UV-1900i, Kyoto, Japan). Test samples were prepared by mixing 1.2 mL of F2–F8 solutions with 1.12 mL of PBS and 80 µL of 1% egg white albumin (Sigma-Aldrich, A5378). Diclofenac sodium (1.5 mg mL^−1^), DMSO (15% *v*/*v* [94]), and distilled water served as the positive, negative, and reference controls, respectively. Test samples, in three replicates, were preincubated at 37 °C for 15 min in a water bath, then heated at 70 °C for 15 min, cooled to room temperature, and centrifuged at 11,000× *g*. The supernatant was then measured at 660 nm. The percentage inhibition of protein thermal denaturation was calculated using the following formula:

(5) $$\%ITPD=\frac{A_{R}-(A_{S}-A_{SN})}{A_{N}}\times 100$$

where

ITPD—inhibition of protein thermal denaturation;

A_R—_absorbance of the reference control;

A_S_—absorbance of the sample after thermal inactivation test;

A_SB_—absorbance of the sample negative control.

### 4.9. Statistical Analysis

Statistical analysis of the experimental data was performed using one-way analysis of variance (ANOVA) followed by Tukey’s HSD post hoc test at a significance level of *p* < 0.05, using Statistica software version 13.3 (TIBCO Software Inc., Palo Alto, CA, USA).

## 5. Conclusions

In this paper, we developed novel poly(isosorbide sebacate)/dimethyl isosorbide (PISEB/DMI) liquid formulations intended for use as in situ forming implants, which can be administered to the target site using a standard syringe and needle. The formulations were loaded with bacteriostatic tetracyclines; however, while the system proved suitable for doxycycline (DOXY), minocycline (MIN) was released too rapidly, suggesting that further optimization of the formulation is required for this particular antibiotic. Eugenol (EUG), included as an additional active agent, significantly influenced the release profile of antibiotics from precipitated depots and enhanced the antibacterial activity of released antibiotics, thereby eliciting an anti-inflammatory response. This study demonstrates that ISFI systems based on PISEB/DMI represent a promising alternative to PLA/PLGA-based depots for localized periodontal therapy.

## Figures and Tables

**Table 1:** Composition of prepared PISEB-based ISFI formulations.

Formulation	Composition *			
	DMI, mg	EUG, mg	DOXY, mg	MIN, mg
F1	1600	-	-	-
F2	1600	-	200	-
F3	1440	160	200	-
F4	1600	-	-	200
F5	1440	160	-	200
F6	1440	160	-	-
F7	1120	480	-	-
F8	800	800	-	200

* All amounts are based on 400 mg of PISEB.

**Table 2:** Release parameters of DOXY (F2), DOXY with EUG (F3), MIN (F4), MIN with EUG (F5), and EUG (F6-F8) based on different kinetic models.

Formulation	Zero-Order Model		First-Order Model		Higuchi Model	
	r^2^	k_0_, h^−1^	r^2^	k_1_, h^−1^	r^2^	k_H_, h^−1/2^
F2	0.7025	0.1193	0.8774	0.00276	0.9548	2.493
F3	0.2911	0.0603	0.3828	0.00092	0.9519	0.574
F4	0.2053	0.0876	0.5693	0.06633	0.5355	0.514
F5	0.2699	0.0861	0.5205	0.00276	0.9310	0.752
F6	0.6225	0.1552	0.9885	0.01865	0.8392	3.404
F7	0.6446	0.1705	0.9790	0.02464	0.8067	3.984
F8	0.6577	0.1704	0.9955	0.01796	0.8088	4.054

**Table 3:** Zones of inhibition obtained with solutions released from F2 and F3 formulations against selected bacterial strains.

Bacteria	1 Day		21 Days	
	F2	F3	F2	F3
*E. coli* ATCC 25922	18.7 ± 1.53 ^a^	22.3 ± 0.58 ^b^	0 ± 0 ^a^	9.33 ± 1.15 ^b^
*S. aureus* ATCC 25923	31.7 ± 1.53 ^a^	37.3 ± 3.06 ^b^	13.0 ± 1 ^a^	22.7 ± 0.58 ^b^
*S. mutans* ATCC 25175	33.3 ± 1.53 ^a^	35.0 ± 0.0 ^a^	0 ± 0 ^a^	19.0 ± 1.73 ^b^
*S. sobrinus* ATCC 33478	31.0 ± 1.0 ^a^	40.0 ± 0.0 ^b^	0 ± 0 ^a^	18.0 ± 1.73 ^b^

The diameters in mm are mean ± SD (n = 3). Different letters (^a,b^) indicate significant differences between F2 and F3 formulations for the same strain on the same day (*p* < 0.05).

## Data Availability

The data can be provided upon request.

## Supplementary Materials

The following supporting information can be downloaded at: https://www.mdpi.com/article/10.3390/molecules30244717/s1, Figure S1: Chemical formulae of the ISFI formulations components, (A) poly(isosorbide sebacate) (PISEB), (B) dimethyl isosorbide (DMI), (C) doxycycline hyclate (DOXY), (D) minocycline hydrochloride (MIN), (E) eugenol (EUG); Figure S2: The release data from (A) F2 and (B) F6 implant plotted in accordance with various kinetic models; Figure S3: The antimicrobial effect of active compouds released from formulation F2 (DOXY) and F3 (DOXY + EUG) after 24 h of experiment; Figure S4: SEM microphotographs presenting the influence of active compounds released from selected formulations on the ability of *S. mutans* and *S. sobrinus* to form a biofilm; Figure S5: ^1^H NMR spectrum (A) and ^13^C NMR spectrum (B) of poly(isosorbide sebacate) (PISEB); Figure S6: DSC first (A) and second (B) heating run of poly(isosorbide succinate) (PISEB).

## Abbreviations

The following abbreviations are used in this manuscript:

ATCC	American Type Culture Collection
CFU	Colony-forming units
DMI	Dimethyl isosorbide
DOXY	Doxycycline hyclate
EUG	Eugenol
IS	Isosorbide
ISFI	In situ forming implants
MIC	Minimum inhibitory concentration
MIN	Minocycline hydrochloride
PBS	Phosphate-buffered solution
PISEB	Poly(isosorbide sebacate)
PLA	Polylactide
PLGA	Poly(lactide-co-glycolide)
