# Supplementary material for: Antibacterial Agent-Loaded, Novel In Situ Forming Implants Made with Poly(Isosorbide Sebacate) and Dimethyl Isosorbide as a Solvent for Periodontitis Treatment
Source: Molecules. 2025 Dec 9;30(24):4717. doi: 10.3390/molecules30244717 (PMC12736199; doi:10.3390/molecules30244717)
Supplement: Supplementary file 1 [file molecules-30-04717-s001.zip › molecules-4023421-supplementary.pdf]

Supplementary Information

"Antibacterial agent-loaded, novel in situ forming implants made with poly(isosorbide sebacate) and dimethyl isosorbide as a solvent for periodontitis treatment"

Monika Śmiga-Matuszowicz*, Bożena Nowak*, Danuta Wojcieszyńska*

*Corresponding authors:

Monika Śmiga-Matuszowicz, Department of Physical Chemistry and Technology of Polymers, Silesian University of Technology, Gliwice,
e-mail: monika.smiga-matuszowicz@polsl.pl

Bożena Nowak, Institute of Biology, Biotechnology and Environmental Protection, Faculty of Natural Sciences, University of Silesia in Katowice
e-mail: bozena.d.nowak@us.edu.pl
Danuta Wojcieszyńska, Institute of Biology, Biotechnology and Environmental Protection, Faculty of Natural Sciences, University of Silesia in Katowice
e-mail: bozena.d.nowak@us.edu.pl

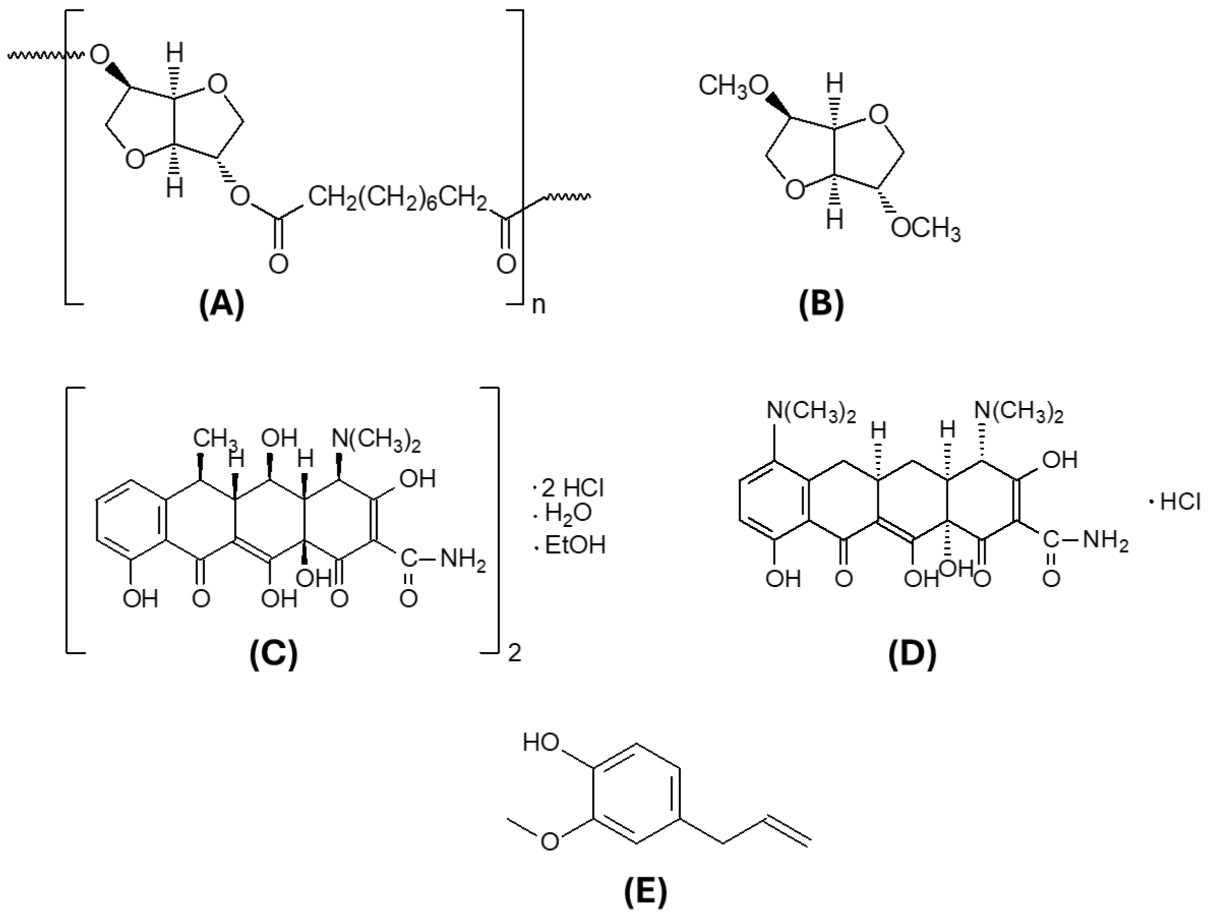

Figure S1. Chemical formulae of the ISFI formulations components, (A) poly(isosorbide sebacate) (PISEB), (B) dimethyl isosorbide (DMI), (C) doxycycline hyclate (DOXY), (D) minocycline hydrochloride (MIN), (E) eugenol (EUG).

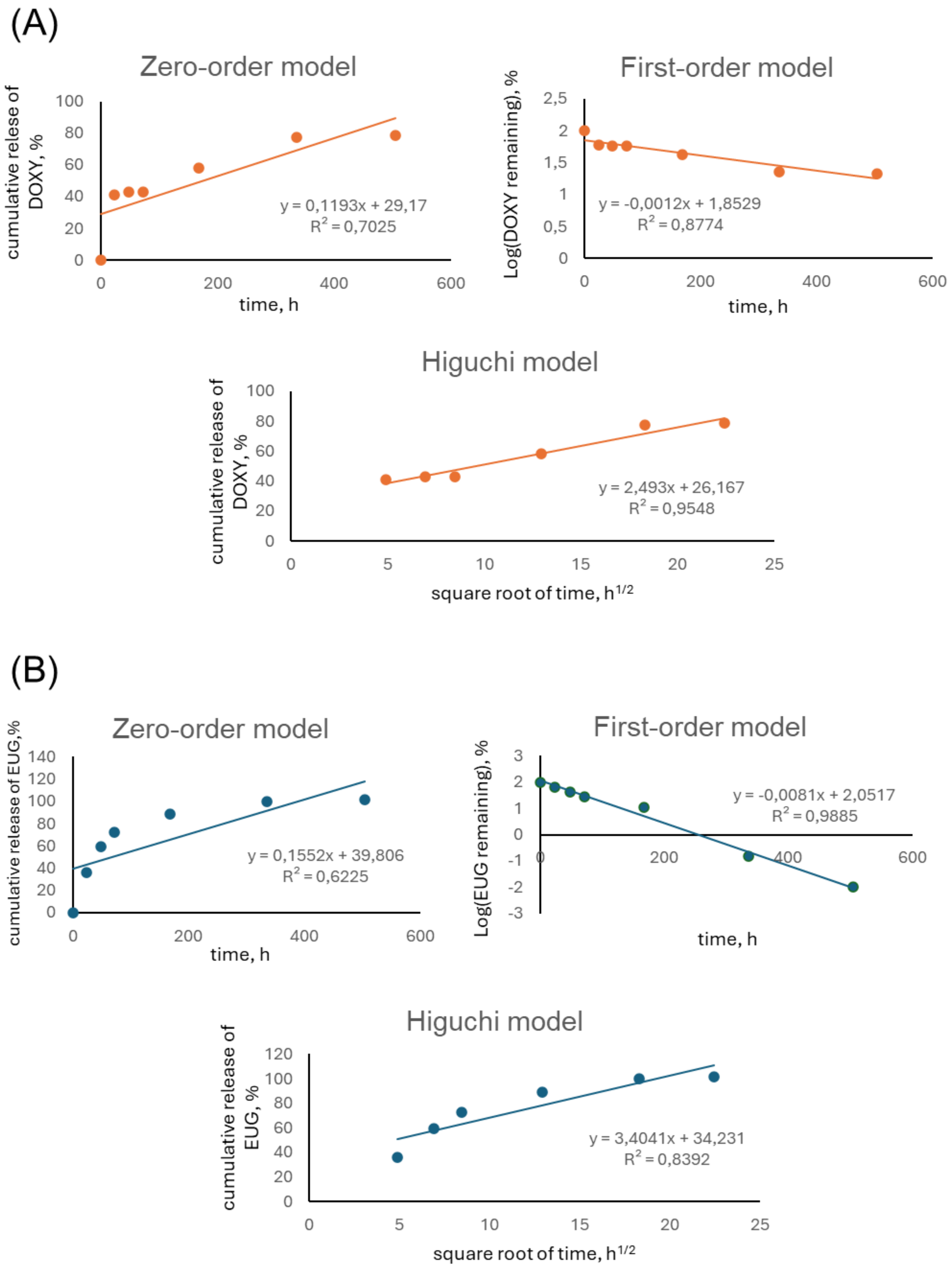

Figure S2. The release data from (A) F2 and (B) F6 implant plotted in accordance with various kinetic models.

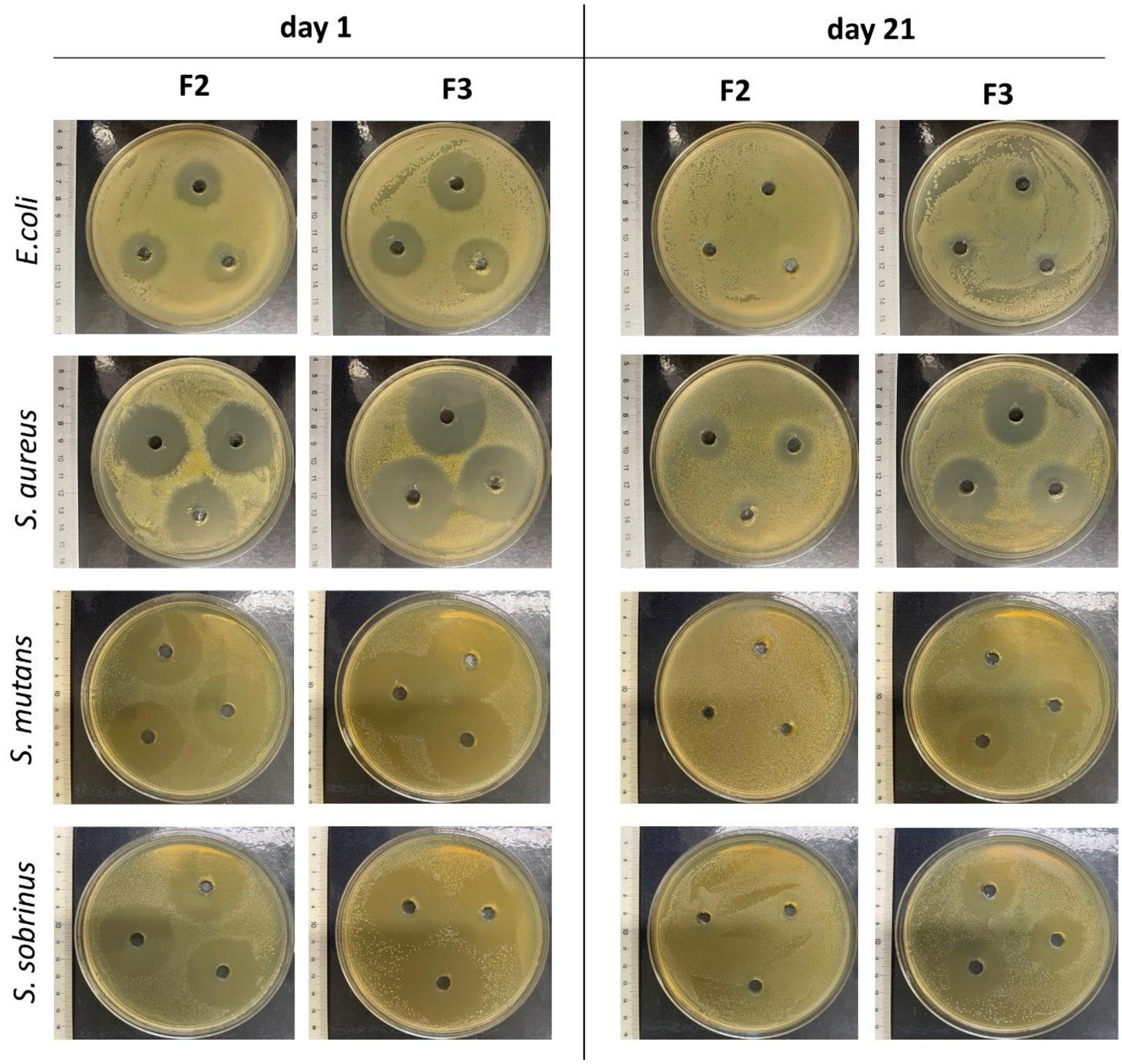

Figure S3. The antimicrobial effect of active compouds released from formulation F2 (DOXY) and F3 (DOXY + EUG) after 24 hours of experiment.

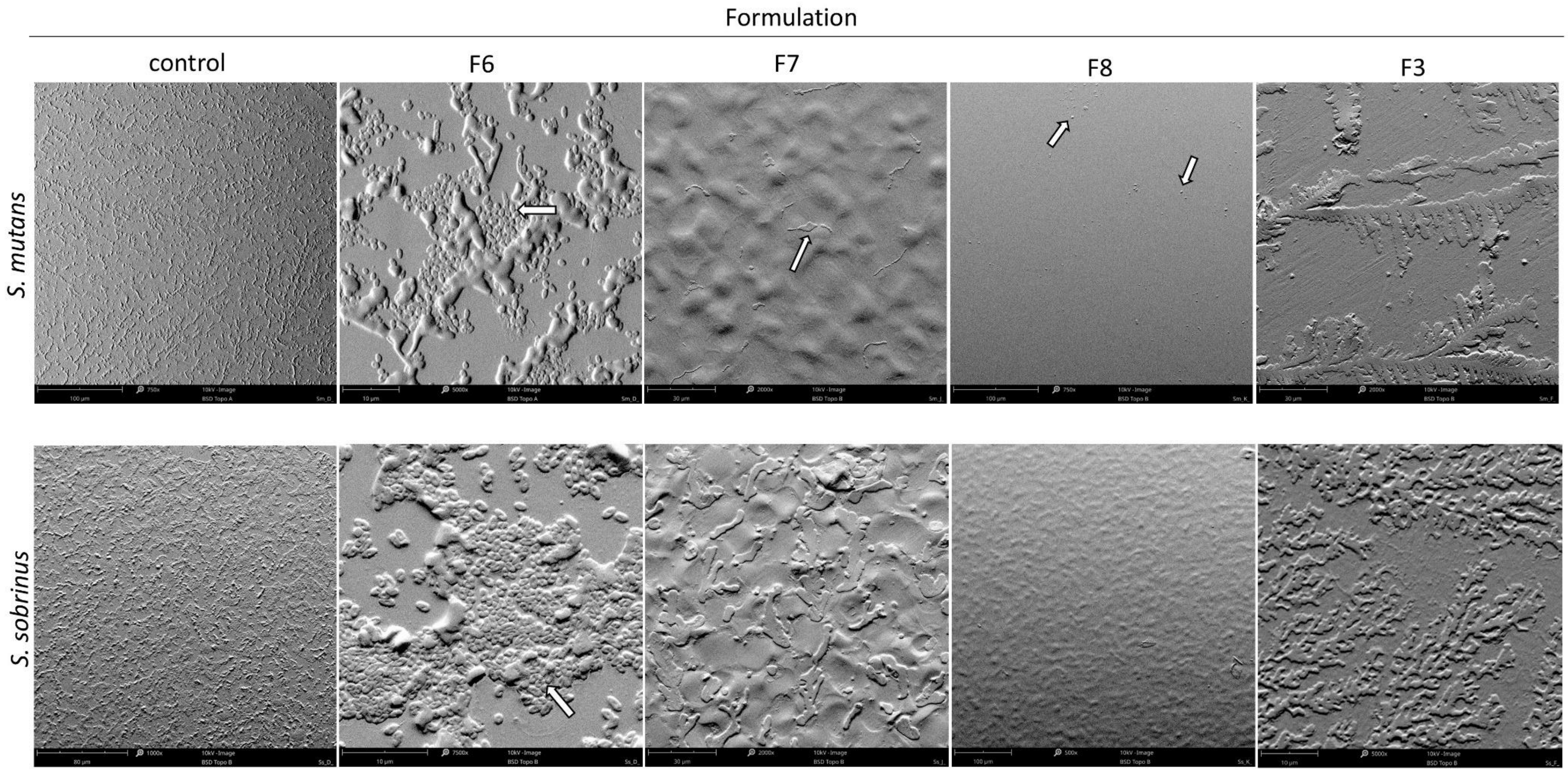

Figure S4. SEM microphotographs presenting the influence of active compounds released from selected formulations on the ability of *S. mutans* and *S. sobrinus* to form a biofilm.

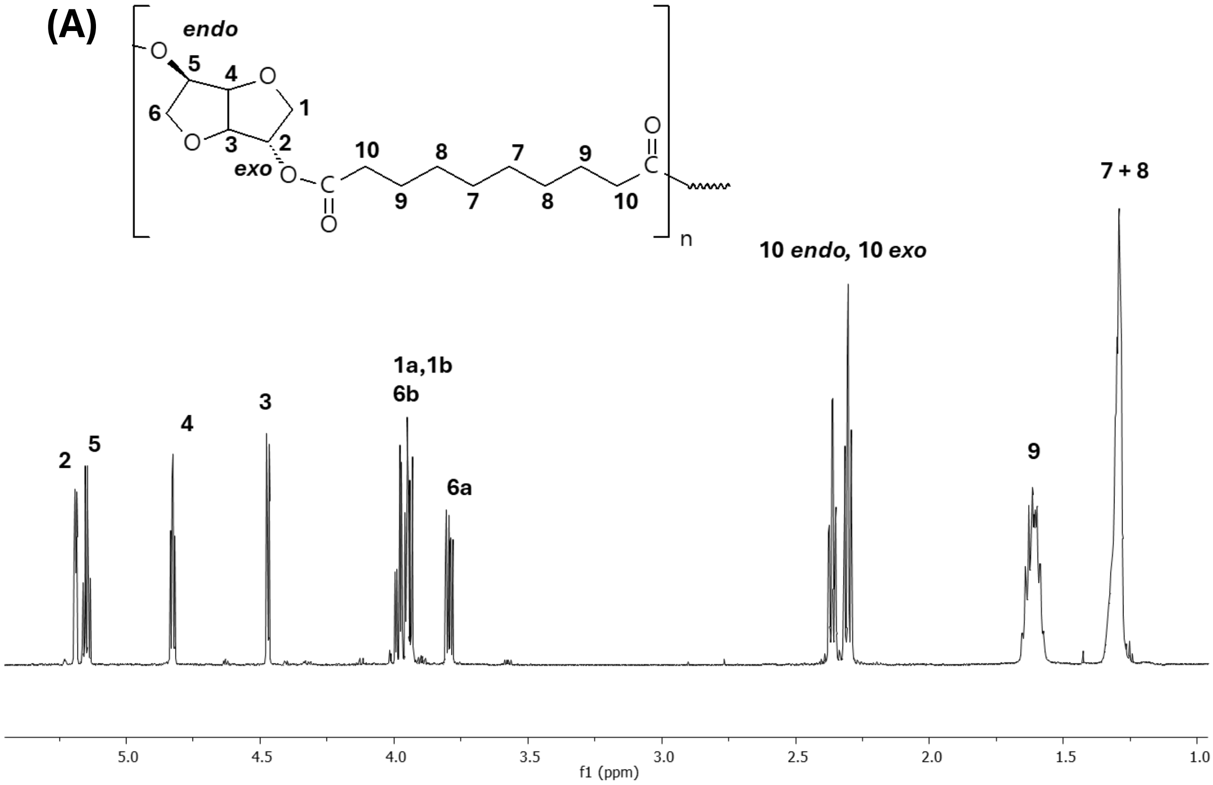

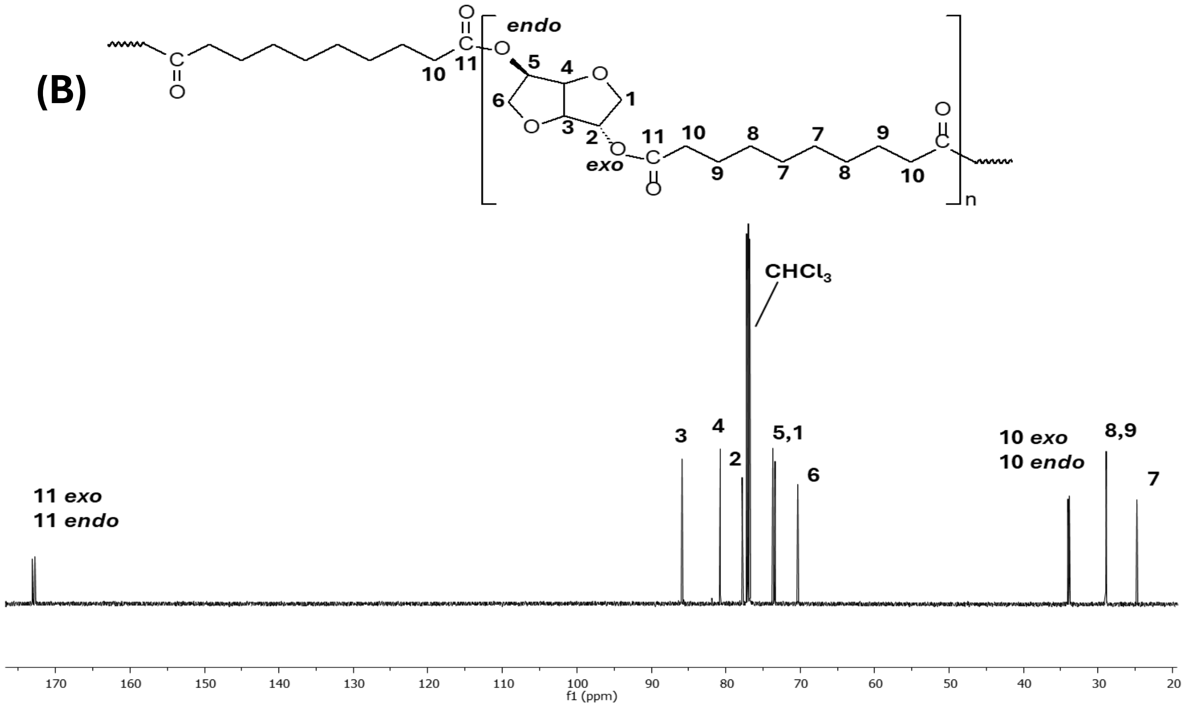

Figure S5. ^{1}H NMR spectrum (A) and ^{13}C NMR spectrum (B) of poly(isosorbide sebacate) (PISEB).

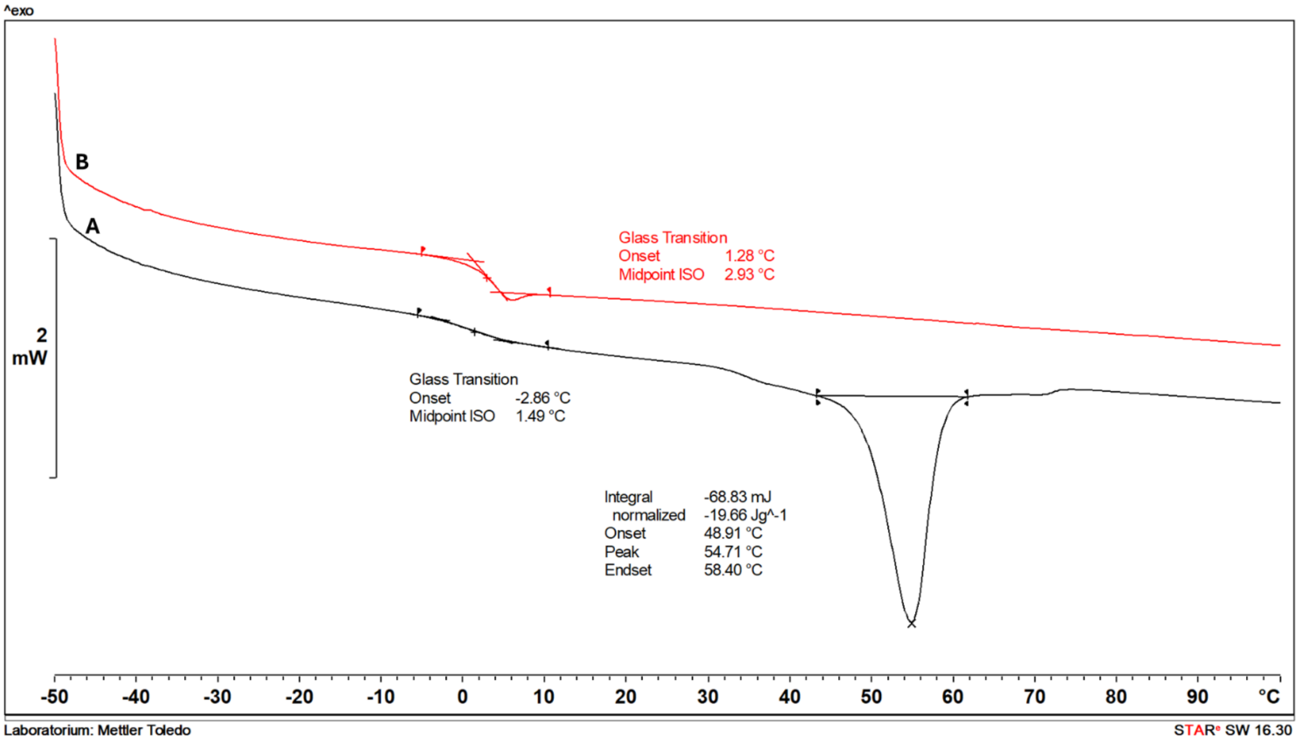

Figure S6. DSC first (A) and second (B) heating run of poly(isosorbide succinate) (PISEB).
